# Counselor value guidance and lower psychological crisis vulnerability: dual meaning-and-resilience pathways in cross-sectional and cluster-randomized evidence from Chinese universities

**DOI:** 10.3389/fpsyg.2026.1881332

**Published:** 2026-07-22

**Authors:** Suye Sun

**Affiliations:** The Second Clinical Medical College, Nanchang University, Nanchang, China

**Keywords:** Chinese university students, cluster-randomized trial, counselor value guidance, meaning in life, psychological crisis vulnerability, psychological resilience

## Abstract

**Introduction:**

Psychological crisis vulnerability among Chinese university students remains an urgent concern, yet protective institutional resources are less well specified than risk pathways. We examined whether counselor value guidance, operationalized as autonomy-supportive counselor relating in a value-relevant context, is associated with lower crisis vulnerability through two complementary mechanisms: meaning construction and resilience building.

**Methods:**

Study 1 was a cross-sectional survey of 800 undergraduates testing parallel mediation and moderation by value internalization. Study 2 was an exploratory cluster-randomized trial across 16 intact classes (400 recruited; 319 complete cases) evaluating a 6-week structured value-guidance workshop against lecture-based mental-health control. Because Study 2 was internally time-stamped but not prospectively registered in a public registry, H3-H4b are framed as exploratory intervention tests guided by Study 1’s a priori model.

**Results:**

In Study 1, counselor value guidance negatively predicted crisis vulnerability, β = −0.373, 95% CI (−0.435, −0.314). Indirect effects through meaning [−0.068, 95% CI (−0.107, −0.031)] and resilience [−0.115, 95% CI (−0.148, −0.085)] were significant. The pathway contrast included zero, and *post-hoc* TOST bounded the difference within ± 0.10 standardized units. Value internalization moderated the meaning pathway [index = −0.022, 95% CI (−0.036, −0.010)]. In Study 2, the Group × Time effect on crisis vulnerability was significant, *F*(2, 634) = 20.48, *p* < 0.001, partial η^2^ = 0.061, with T2 *d* = 0.43 and T3 *d* = 0.37. Intervention-induced changes in meaning and resilience both mediated vulnerability change, with nearly identical indirect effects (−0.036 and −0.035).

**Conclusion:**

Across correlational and exploratory intervention evidence, counselor value guidance was associated with lower crisis vulnerability, with meaning and resilience functioning as parallel statistical pathways of comparable order of magnitude. Publicly preregistered replications with longer follow-up are needed before the parallel-magnitude pattern is treated as confirmatory.

## 1 Introduction

Psychological crisis vulnerability refers to the extent to which individuals lack the capacity to withstand crises arising from sudden events or major setbacks (Guo et al., 2018). Among Chinese university students, mental-health problems have shown persistent or increasing trends over the past decade (Chen et al., 2022; Lin et al., 2025), and existing research has documented several risk pathways: ostracism predicts elevated crisis vulnerability through self-uncertainty and subjective social status (Li and Li, 2026); social exclusion increases suicide intention through depression (Zhu et al., 2024); and depression and ostracism associate reciprocally via interpretation bias (Bar-Sella et al., 2022). Far less empirical attention has been paid to institutional protective resources that might mitigate vulnerability.

In Chinese higher education, the counselor (Fu dao yuan) is a full-time staff member assigned to each class at approximately 1:200. The role combines functions distributed across academic advising, residence-life coordination, and frontline mental-health gatekeeping in Western universities, with weekly to biweekly student contact and substantive engagement in value-oriented dialogue. Recent implementation work such as the ACE-LYNX intervention for Chinese university health-service providers (Wang F. et al., 2024) shows that structured training of frontline staff can improve service quality, but the underlying pathways through which value-oriented counselor guidance protects student mental health remain underspecified.

Against this background, three gaps motivate the present research. First, protective institutional resources—and the Chinese counselor role in particular—remain far less specified empirically than risk factors, even though the counselor system is structurally positioned to influence student wellbeing. Second, the mechanisms through which counselor guidance might relate to lower vulnerability are largely untested: prior implementation work demonstrates that training frontline staff can improve service quality (Wang F. et al., 2024) but does not identify the intermediate psychological processes involved. Third, no prior study has examined whether meaning-based and resilience-based routes contribute comparably or asymmetrically to this protective association—the equal-magnitude question (H2c) that is one of the principal contributions of this work. The present two-study design targets all three gaps: Study 1 specifies and tests the dual-pathway architecture correlationally, and Study 2 provides an exploratory intervention test of whether the same two pathways can be engaged.

We propose a dual-pathway protective model in which perceived counselor value guidance is hypothesized to relate to lower crisis vulnerability through (a) meaning construction, grounded in Self-Determination Theory (SDT; Deci and Ryan, 2000; Ryan and Deci, 2017; Ryan et al., 2022), and (b) resilience building, grounded in Conservation of Resources theory (COR; Hobfoll, 1989, 2002). SDT emphasizes internal value coherence and meaning-construction processes that follow from autonomy-supportive social contexts; COR emphasizes the accumulation of psychological resources that buffer stress. The two theories are theoretically complementary, but they do not, on their face, predict equal protective magnitude. Whether the two routes contribute equally or asymmetrically to the protection of vulnerability is the empirical question H2c pre-specified in the present study, and is one of our principal contributions to the literature.

### 1.1 Theoretical background

The dual-pathway model integrates two complementary motivational and resource theories. Self-Determination Theory (SDT; Deci and Ryan, 2000; Ryan and Deci, 2017; Ryan et al., 2022) holds that autonomy-supportive social contexts—those that offer choice, acknowledge perspectives, and minimize external control—facilitate the internalization of values and, in turn, a more coherent and self-endorsed sense of meaning. Applied to the counselor context, autonomy-supportive value guidance is expected to support students’ construction of personal meaning, which SDT links to wellbeing and reduced distress. This grounds the meaning-construction pathway (Pathway 1) and motivates the moderation prediction (H2d): students who have more fully internalized the values discussed with their counselor (higher SRQ-I) should show a stronger meaning-related indirect association, because internalization is the mechanism through which autonomy support is theorized to translate into meaning.

Conservation of Resources theory (COR; Hobfoll, 1989, 2002) holds that individuals seek to acquire, retain, and protect valued resources, and that resource reservoirs buffer the impact of stressors and reduce vulnerability to crisis. Supportive relationships are a key conduit for resource gain (Halbesleben, 2006), and psychological resilience can be understood as an accumulated personal resource that protects functioning under adversity (Egozi Farkash et al., 2022). Applied to the counselor context, value-relevant counselor support is expected to contribute to students’ resilience resources, which in turn buffer crisis vulnerability. This grounds the resilience-building pathway (Pathway 2).

Although SDT and COR are conceptually complementary—one emphasizing meaning that follows from internalized values, the other emphasizing resources that buffer stress—they do not, on their face, predict that the two routes should be of equal protective magnitude. SDT-based accounts might privilege the meaning route in contexts emphasizing autonomy-supportive value transmission, whereas COR-based accounts might privilege the resilience route in contexts dominated by resource-loss pressures. Whether the two indirect associations are of comparable magnitude in the present institutional context is therefore a genuinely open theoretical question, which we formalize as the a priori equal-magnitude prediction (H2c) and examine in both studies.

A construct-validity caveat warrants stating up front. The instrument we use to operationalize counselor value guidance (the 20-item Counselor Value Guidance Scale, CVGS; described in section 2.4) is an extension of the LCQ autonomy-support tradition (Williams and Deci, 1996), and several of its item wordings remain closely aligned with autonomy-supportive relating (e.g., providing choice, encouraging questions) rather than directly indexing value content or value modeling. The empirical effects we document throughout therefore most rigorously characterize an autonomy-supportive counselor-student relating effect in a value-relevant institutional context, with “value guidance” functioning as a context label rather than as the principal item content. We return to this distinction in sections 2.4 and 4.3.

To test this model, we conducted two complementary studies. Study 1 used a cross-sectional design (*N* = 800) to test the parallel-mediation architecture and a moderated-mediation extension involving value internalization (H2d). Study 2 used an exploratory cluster-randomized trial (16 classes, *N* = 319 completers) to examine whether a structured 6-week value-guidance workshop could engage the dual pathway, with separate manipulation-check indicators for each pathway prerequisite. Consistent with Fischer et al.’s (2025) measurement-invariance-as-mapping perspective, we treat counselor value guidance as a culturally specific operationalization of broader autonomy-supportive mentoring and meaning-centered education traditions (Steger, 2009; Williams and Deci, 1996), referring to value clarification and meaning construction rather than politically directive content.

The hypotheses tested in the present research are summarized below; full hypothesis statements are reported in section 2.1.

*H1*: Perceived counselor value guidance is negatively associated with psychological crisis vulnerability.

*H2a/H2b*: Meaning in life and resilience each significantly mediate the association in H1.

*H2c*: The two indirect effects are of comparable magnitude (equal-magnitude prediction; pre-specified).

*H2d*: Value internalization (SRQ-I) moderates the meaning pathway, with stronger indirect effects at higher internalization.

*H3* (exploratory Study 2 extension): A 6-week structured value-guidance workshop reduces crisis vulnerability at T2 (post) and T3 (8-week follow-up).

*H4a/H4b* (exploratory Study 2 extensions): Intervention-induced changes in meaning and resilience each mediate the protective effect on vulnerability change.

**FIGURE 1:**
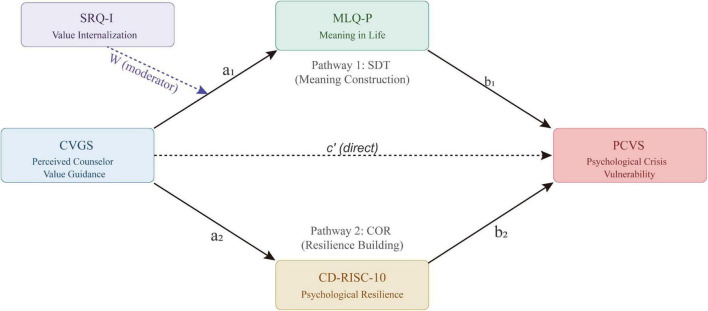
Hypothesized dual-pathway protective model. Perceived counselor value guidance (operationalized via the 20-item Counselor Value Guidance Scale, CVGS, a context-specific extension of the Learning Climate Questionnaire, LCQ; Williams and Deci, 1996) is hypothesized to reduce psychological crisis vulnerability (PCVS) both directly (path c’) and indirectly through two parallel mediators: Pathway 1 reflects an SDT-grounded meaning-construction route through Meaning in Life (MLQ-P; paths a_1_, b_1_), and Pathway 2 reflects a COR-grounded resilience-building route through psychological resilience (CD-RISC-10; paths a_2_, b_2_). Value internalization (SRQ-I) is hypothesized to moderate the a1 path (W). For construct-validity considerations regarding the LCQ-to-CVGS extension see sections 1, 2.4, and 4.3.

The hypothesized dual-pathway protective model is presented in Figure 1.

## 2 Materials and methods

### 2.1 Hypotheses and design

Two studies were conducted. Study 1 was a cross-sectional dual-pathway mediation study with moderation by value internalization, testing H1-H2d. Study 2 was a cluster-randomized controlled intervention with three measurement points (T1 baseline, T2 immediate post-intervention, T3 8-week follow-up), evaluating H3-H4b as exploratory intervention extensions of the Study 1 model. The analytic plan and Study 2 randomization seed were documented internally before data collection, but the trial was not prospectively registered in a public third-party registry. We therefore use “a priori specified” for Study 1 and H2c, and “exploratory” for the Study 2 intervention hypotheses. A future full-scale replication will be prospectively registered before recruitment.

### 2.2 Study 1: participants and procedure

Of 1,000 Chinese undergraduates invited from two universities, *N* = 800 valid analytic cases were retained after pre-specified quality screening (attention-check failure, straight-line responding, completion-time outliers; exclusion rate 20.0%). Participants were full-time undergraduates aged 18 years or older, gave informed consent in Mandarin, and received 30 RMB for completion. The observed age range was 18–25 years (*M* = 20.02, SD = 1.36) (Table 1). Study 1 and Study 2 used independent cohorts from the same two universities, verified by anonymized enrollment-ID matching. The CFA measurement model was first estimated on the *n* = 204 cases with complete data on all 61 indicator items so that all fit indices used a common observed covariance matrix; an item-level FIML CFA sensitivity check using all available indicators is reported in section 3.1.1 and did not change the measurement conclusion. Substantive regression and mediation analyses used the full *N* = 800 with pairwise-available indicators or FIML where indicated. Across the full sample (*N* = 800), 204 cases had complete data on all 61 indicators and 364 cases showed at least one missing item; the item-level missing rate was approximately 2.26%, and FIML was used to retain all available cases in the sensitivity CFA and substantive models.

**TABLE 1:** Sample characteristics for Study 1 (*N* = 800) and Study 2 by allocation arm (intervention *n* = 157; control *n* = 162).

Characteristic	Category	Study 1 total	Study 2 intervention	Study 2 control
Sample size	N	800	157	162
Age	M (SD), range	20.02 (1.36), 18–25	19.94 (1.15), 18–23	19.86 (1.19), 18–23
Gender	Male	336 (42.0%)	62 (39.5%)	77 (47.5%)
Gender	Female	454 (56.8%)	93 (59.2%)	84 (51.9%)
Gender	Other/unspecified	10 (1.2%)	2 (1.3%)	1 (0.6%)
Grade	Year 1	211 (26.4%)	46 (29.3%)	65 (40.1%)
Grade	Year 2	226 (28.2%)	51 (32.5%)	45 (27.8%)
Grade	Year 3	213 (26.6%)	36 (22.9%)	33 (20.4%)
Grade	Year 4	150 (18.8%)	24 (15.3%)	19 (11.7%)
Major type	Humanities/social science	369 (46.1%)	78 (49.7%)	78 (48.1%)
Major type	STEM/medical/agricultural	374 (46.8%)	70 (44.6%)	72 (44.4%)
Major type	Other/interdisciplinary	57 (7.1%)	9 (5.7%)	12 (7.4%)
Urban/rural origin	Urban	367 (45.9%)	63 (40.1%)	74 (45.7%)
Urban/rural origin	Town	202 (25.2%)	50 (31.8%)	44 (27.2%)
Urban/rural origin	Rural	231 (28.9%)	44 (28.0%)	44 (27.2%)
Only child	Yes	372 (46.5%)	85 (54.1%)	59 (36.4%)
Only child	No	428 (53.5%)	72 (45.9%)	103 (63.6%)
School type	Elite	287 (35.9%)	77 (49.0%)	83 (51.2%)
School type	Regular	513 (64.1%)	80 (51.0%)	79 (48.8%)

Percentages are within-column. Age is reported as M (SD), range. Study 1 was a cross-sectional sample; Study 2 was a cluster-randomized trial with 16 intact classes (8 per arm). School type elite vs. regular refers to institutional tier; both studies recruited from the same two universities but used independent participant pools (no overlap; see sections 2.2 and 2.3). The only-child distribution differs significantly between the Study 2 arms (54.1% intervention vs. 36.4% control, χ^2^(1) = 9.41, *p* = 0.002); this baseline imbalance is statistically adjusted in the primary analyses and is discussed in sections 3.2.1 and 4.3.

### 2.3 Study 2: design, intervention, and participants

Sixteen intact classes from two universities (8 intervention, 8 control) were block-randomized to condition in R, with the random seed time-stamped before T1. Reporting follows the CONSORT 2010 extension for cluster-randomized trials (Campbell et al., 2012), and recent school-based mental-health CRTs are used as reporting comparators where relevant (e.g., Grummitt et al., 2025; Kuyken et al., 2022). Eligibility criteria were identical to Study 1; all randomized participants were legal adults (observed age range 18–23 years) (Table 1). Randomization was performed by an investigator independent of recruitment and outcome assessment, and class allocations were released only after baseline data had been collected and de-identified. Participants and facilitators could not be blinded because the workshop and lecture formats were overtly different, but survey administration, data de-identification, preliminary QC, baseline checks, ICC computation, and attrition coding were performed before analysts were unblinded to the cluster-condition mapping. Participants received 30 RMB per completed wave and could withdraw without losing compensation for completed waves. Recruitment yielded *N* = 400 students; 373 completed T2 and 337 completed T3. After uniform T3 quality-control screening, the final complete-case analytic sample was *N* = 319 (162 control, 157 intervention), reflecting 15.8% attrition plus 4.5% QC exclusion. ITT-LOCF, FIML, per-protocol, leave-one-facilitator-out, and fidelity-weighted sensitivity analyses are reported in section 3.2.5.

G*Power calculations for a 2 × 3 mixed ANOVA (α = 0.05, 1 −β = 0.80, partial η^2^ = 0.04, within-subject *r* = 0.50) required minimum *N* = 74; Monte Carlo simulations for difference-score mediation required *N* ≥ 250 (Schoemann et al., 2017); design-effect correction used the analytic cluster size of approximately 20 with assumed ICC = 0.05 (design effect = 1 + (20 − 1) × 0.05 = 1.95), yielding effective *N* ≈ 164. The analytic target *N* ≈ 320 accommodates all constraints with attrition margin; the actual realized final analytic sample was *N* = 319.

The intervention was a 6-week structured workshop (6 weekly 90-min sessions) delivered by eight trained facilitators, one per intervention class. The 6-week dosage was not intended to durably build trait resilience, which develops over far longer time frames; rather, it was designed as an exploratory, proof-of-concept test of whether the hypothesized meaning- and resilience-related processes could be engaged sufficiently to produce short-term change in crisis vulnerability and to mediate that change. This framing is reflected in the manipulation-check and mediation analyses and is revisited in section 4.3. Sessions covered value clarification, meaning-construction exercises, resilience-skills training, and value-meaning reflection homework. The control condition received six 90-min lectures on general mental-health topics delivered by separate control-pool facilitators. Facilitators were nested within condition and received weekly supervision. Workshop fidelity was measured only in the intervention arm because the lecture control had no equivalent checklist by design; fidelity was treated as a process indicator, with no pre-specified rule for excluding or adjusting participants or clusters on the basis of fidelity. We therefore report a *post-hoc* fidelity-weighted sensitivity analysis rather than using fidelity to define the primary estimand. Participant safety procedures flagged high PCVS scores or active self-harm/suicidal ideation within 48 h and offered referral through the university counseling pathway. Across the study window, 31 participant-wave events were flagged (17 intervention, 14 control), five participants initiated counseling-center contact, no hospitalization or emergency mental-health admission was recorded, and no flagged participant withdrew after outreach.

### 2.4 Measures

Counselor value guidance was operationalized as the 20-item Counselor Value Guidance Scale (CVGS), a context-specific extension developed within the SDT autonomy-support tradition operationalized by the Learning Climate Questionnaire (LCQ; Williams and Deci, 1996), itself adapted from the Health Care Climate Questionnaire (Williams et al., 1996). Whereas the original 15-item LCQ was designed to assess autonomy-supportive climate in instructor–student or physician–patient relational contexts, the CVGS was extended to 20 items organized around four intended content-area categories (value-clarification guidance, meaning-construction facilitation, modeling/demonstration, and resilience-building support) referencing the assigned counselor (e.g., “My counselor provides me choices and options”; “My counselor encourages me to ask questions about the values we discuss”), rated on a 7-point scale. We treat these four categories as a priori-specified content-organizational categories used during item generation rather than as empirically validated orthogonal facets; their empirical separability under CFA is examined in section 3.1.1, where we report comparisons with a unidimensional model and with a higher-order autonomy-support model. The mean of all items served as the total CVGS score, with higher scores indicating stronger perceived value guidance. The CVGS was developed for the present research; broader psychometric validation (cross-sample replication of any facet structure, measurement invariance, test–retest reliability) is identified as a limitation in section 4.3. The CVGS was generated for the present study without a separate pilot study or formal expert content-validity review (e.g., a content-validity-index procedure); item generation was guided by the LCQ autonomy-support tradition and the four a priori content-area categories described above. Psychometric evaluation in this paper is therefore limited to the present-sample CFA and reliability estimates, and the absence of pilot testing and expert review is acknowledged as a limitation in section 4.3.

A construct-validity point also warrants explicit attention here. Although the CVGS is organized around four intended content-area categories (section 2.4) meant to span the value-guidance space, several item wordings remain close to the parent LCQ tradition. The construct measured by total CVGS scores in this study is therefore best interpreted as autonomy-supportive counselor relating in a value-relevant context, rather than as value transmission proper. We retain the name “Counselor Value Guidance Scale” for continuity with the institutional context within which it was developed, but section 3.1.1 reports that the four content-area categories are not strongly differentiated as orthogonal facets in the present CFA evidence, and section 4.3 discusses scale-refinement directions that would tighten the alignment between scale name, item content, and facet structure.

Other measures (Chinese versions verified by Brislin, 1970, back-translation): Meaning in Life Questionnaire–Presence subscale (MLQ-P; Steger et al., 2006; Liu and Gan, 2010): 5 items, 7-point Likert scale (1 = strongly disagree to 7 = strongly agree). Connor-Davidson Resilience Scale (CD-RISC-10; Campbell-Sills and Stein, 2007; Wang et al., 2010): 10 items, 5-point Likert scale (0 = not true at all to 4 = true nearly all the time), total range 0–40. Psychological Crisis Vulnerability Scale (PCVS; Guo et al., 2018): 22 items in four dimensions (Challenge, Coping, Support, Resilience), 5-point Likert scale (1 = strongly disagree to 5 = strongly agree). The PCVS Resilience subscale differs from the CD-RISC-10 mediator in directionality (deficit vs. resource), breadth (crisis-specific vs. trait-general), and embedding (6-item facet vs. 10-item stand-alone); empirical discriminant validity is reported in section 3.1. Psychological Vulnerability Scale (PVS; Sinclair and Wallston, 1999): 6 items, 5-point Likert scale (1 = does not describe me at all to 5 = describes me very well), used as convergent-validity marker. Self-Regulation Questionnaire–Internalization (SRQ-I; Ryan and Connell, 1989): 4 items measuring internalized value endorsement, 5-point Likert scale (1 = not at all true to 5 = very true).

Study 2 included three manipulation-check indicators administered at T2. To avoid abbreviation overlap with the Psychological Vulnerability Scale (PVS) used in Study 1, the input-side check on value-clarification content is reported as the Personal Value-Clarity check (MC-1; hereafter PVC-MC1; 6 items rated on a 5-point Likert scale, 1 = not at all clear to 5 = completely clear, possible range 1–5 averaged across items). The Meaning Reflection Recall Task (MC-3; prerequisite check for the meaning pathway) was an open-ended task scored on 5 content dimensions for depth and accuracy, each dimension rated 0–5, possible range 0–25. The Resilience Skills Comprehension Check (MC-4; prerequisite check for the resilience pathway) consisted of 5 scenario-based items, each scored 0–4 for correct application of taught skills, possible range 0–20. A homework-quality indicator (MC-2; researcher-rated weekly homework on a 0–10 rubric averaged across 6 weeks, possible range 0–10) was administered within the intervention arm only and is reported as an internal fidelity benchmark rather than a between-condition test, since the control condition received lectures with no equivalent homework. Open-ended MC-3 and scenario-based MC-4 responses were each independently scored by two coders, with inter-rater agreement of Cohen’s κ = 0.785 (this coding reliability is distinct from the intervention-arm fidelity video-coding reliability of κ = 0.812 reported in section 3.2.2). Coders were not formally blinded to condition; because intervention-taught content can be identifiable in open-ended responses, scoring blindness could not be fully ensured. This is noted as a limitation in section 4.3.

### 2.5 Statistical analysis

Study 1 analyses used PROCESS Model 4 (parallel mediation) for H1-H2c and Model 7 (moderated mediation on the meaning pathway) for H2d, with 5,000 bootstrap iterations and 95% bias-corrected CIs (Hayes, 2018). H2c was tested as the bootstrap CI of the contrast between the two indirect effects; a CI excluding zero would reject the equal-magnitude prediction. Confirmatory factor analysis in lavaan tested the 11-factor measurement model and three competing CVGS specifications. The primary CFA used the listwise-complete item covariance matrix to keep all 61 indicators and fit indices on the same case base; because other substantive models used FIML where appropriate, a FIML CFA sensitivity analysis was also run and is summarized in section 3.1.1. Common-method-variance diagnostics included Harman’s single-factor PCA and an unmeasured-latent-method-construct model. Sensitivity analyses included serial mediation and FIML estimation.

Study 2 analyses combined mixed ANOVA and linear mixed-effects models. The pre-specified primary analysis was a linear mixed-effects model (LMM) estimated on the intention-to-treat sample (all *N* = 400 randomized participants) with FIML for missing data, following CONSORT guidance for cluster-randomized trials; the mixed ANOVA is reported as a supplementary design-simplified omnibus summary. The complete-case analysis (*N* = 319), per-protocol analysis, ITT-LOCF, leave-one-facilitator-out, and fidelity-weighted analysis (section 3.2.5) are treated as sensitivity analyses around this primary estimand. ITT with FIML was chosen as primary in order to preserve the randomized comparison and to minimize bias from condition-asymmetric attrition. Group × Time omnibus F-tests on PCVS, MLQ-P, and CD-RISC-10 were computed via afex::aov_car(); Greenhouse-Geisser corrections were checked and epsilon values exceeded 0.95, so uncorrected F values are reported. Linear mixed-effects models in lme4 used random intercepts for class, facilitator, and subject, with facilitator nested within condition, and controlled for baseline outcome values and the only-child imbalance in robustness checks. Difference-score mediation of intervention effects on vulnerability change was tested in lavaan with cluster-bootstrap standard errors (cluster = class, 5,000 iterations). Sensitivity analyses included ITT-LOCF, per-protocol analysis ( ≥ 4 of 6 sessions), leave-one-facilitator-out, FIML across all randomized participants, and a *post-hoc* fidelity-weighted analysis in which intervention participants were weighted by class-level fidelity and controls retained unit weight. Manipulation checks were reported as between-condition Welch *t*-tests at T2. These between-condition manipulation-check *t*-tests did not explicitly model the class-level clustering; given the low baseline ICCs ( ≤ 0.055; section 3.2.1), the resulting design effect is small and the impact on these comparisons is expected to be minimal.

## 3 Results

### 3.1 Study 1 results

#### 3.1.1 Measurement model, reliability, and common-method bias

The 11-factor CFA measurement model was estimated first on the *n* = 204 cases with complete data on all 61 indicator items. Listwise deletion was used only for this CFA so that fit indices were based on a single observed covariance matrix; because this choice differs from the FIML approach used in several substantive analyses, we re-estimated the CFA with item-level FIML as a sensitivity check. The FIML CFA yielded the same substantive pattern of fit comparable to, and on the incremental indices somewhat better than, the listwise solution, with all intended factor loadings significant [FIML CFA on the full sample, *N* = 800: χ^2^(1,714) = 2,430.13, CFI = 0.937, TLI = 0.932, RMSEA = 0.023, SRMR = 0.040; standardized loadings 0.381–0.770], and no stronger empirical separation of the four CVGS content categories; therefore, we retain the listwise CFA as the transparent primary measurement model while noting that missing-data handling does not alter the measurement conclusion. In the primary CFA, fit was mixed, with good absolute-fit indices but incremental-fit indices falling just below the conventional 0.90 threshold (Brown, 2015), indicating marginal incremental fit: χ^2^(1,714) = 2,076.0, CFI = 0.891, TLI = 0.883, RMSEA = 0.032, SRMR = 0.060. Standardized item loadings ranged from 0.381 to 0.770, with several CD-RISC-10 items at the lower end of this range; we therefore interpret the measurement model as adequate but not strong. The four-factor CVGS model and the higher-order model fit comparably (Δ CFI within 0.005, Δ RMSEA within 0.001), and the four first-order facets were strongly inter-correlated (latent correlations 0.78–0.91, mean 0.85). We therefore use the 20-item CVGS mean as a higher-order construct in downstream analyses and avoid over-interpreting the facets as distinct constructs. Response-style diagnostics indicated low risk of careless responding, and CMV diagnostics did not indicate a substantive single-source method threat: Harman’s first factor accounted for 18.79% of the variance, and adding an unmeasured latent method construct produced ΔCFI = 0.015 and Δ RMSEA = −0.002 relative to the trait-only model.

Internal consistency in the present sample was acceptable to excellent for the four focal multi-item scales but lower for the brief SRQ-I (Table 2): CVGS Cronbach’s α = 0.907, McDonald’s ω = 0.919; MLQ-P α = 0.837, ω = 0.885; CD-RISC-10 α = 0.745, ω = 0.813; PCVS α = 0.893, ω = 0.908; SRQ-I α = 0.653, ω = 0.794. The four primary scales reached or exceeded the conventional acceptability threshold of α ≥ 0.70 (CD-RISC-10 marginally so); the SRQ-I fell below the α threshold, although its ω approached it. Results involving SRQ-I (notably H2d) are therefore reported with the qualification that the SRQ-I measurement is brief and its α-based reliability below conventional thresholds.

**TABLE 2:** Internal consistency of focal scales (Study 1, *N* = 800).

Scale	Items	α	ω
CVGS	20	0.907	0.919
MLQ-P	5	0.837	0.885
CD-RISC-10	10	0.745	0.813
PCVS	22	0.893	0.908
SRQ-I	4	0.653	0.794
PVS	6	0.710	0.806

CVGS, Counselor Value Guidance Scale; MLQ-P, Meaning in Life Questionnaire–Presence; CD-RISC-10, 10-item Connor-Davidson Resilience Scale; PCVS, Psychological Crisis Vulnerability Scale; SRQ-I, Self-Regulation Questionnaire–Internalization; PVS, Psychological Vulnerability Scale (used as a convergent-validity marker in Study 1).

#### 3.1.2 Descriptive statistics and correlations

Means, standard deviations, and Pearson correlations among focal variables are presented in Table 3. The CVGS mean (*M* = 3.30, SD = 0.56 on a 1–7 scale) fell below the scale midpoint of 4.0, indicating that students on average perceived only modest levels of counselor value guidance—a substantive observation revisited in section 4.2. The SRQ-I mean (*M* = 3.38, SD = 0.64 on the 1–5 scale) fell slightly above the scale midpoint of 3.0, indicating modest above-midpoint endorsement of internalized value motivation. CVGS was negatively correlated with crisis vulnerability (*r* = −0.379, *p* < 0.001) and positively correlated with both mediators (meaning *r* = 0.503; resilience *r* = 0.358). The two mediators were moderately correlated (*r* = 0.279), supporting their treatment as related but nonredundant mechanisms; both correlated negatively with PCVS (meaning *r* = −0.326; resilience *r* = −0.436), with the resilience–PCVS correlation slightly stronger numerically than the meaning–PCVS correlation in this sample.

**TABLE 3:** Means, standard deviations, and zero-order correlations among focal variables (Study 1, *N* = 800).

Variable	*M*	SD	1	2	3	4	5	6
1. CVGS	3.30	0.56	−	−	−	−	−	−
2. MLQ-P	4.72	0.89	0.50**					
3. CD-RISC-10	23.90	5.43	0.36**	0.28**				
4. PCVS	2.91	0.55	−0.38**	−0.33**	−0.44**			
5. PVS	2.83	0.64	−0.26**	−0.21**	−0.26**	0.50**		
6. SRQ-I	3.38	0.64	0.26**	0.30**	0.14**	−0.16**	−0.15**	

PVS, Psychological Vulnerability Scale. **p < 0.01.

#### 3.1.3 Mediation, contrast, and moderated mediation

**FIGURE 2:**
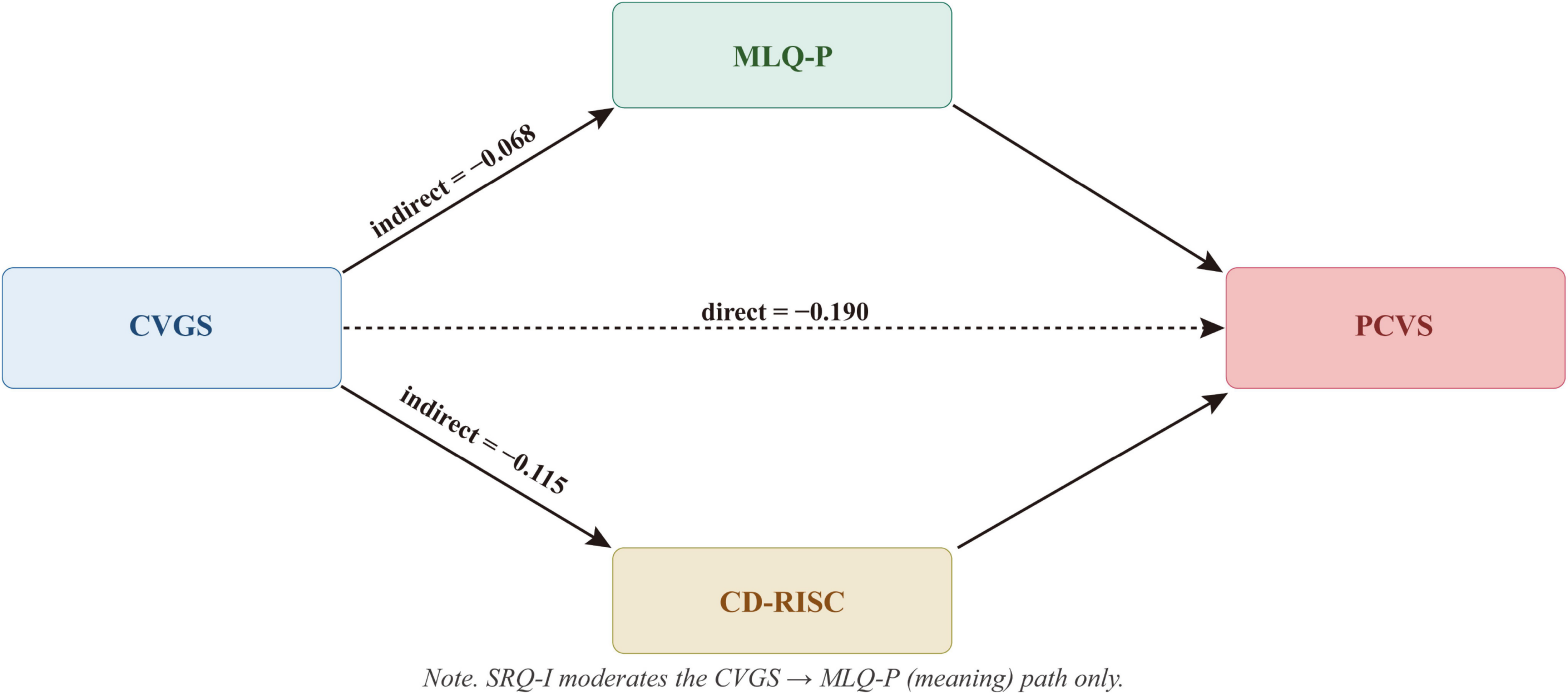
Study 1 dual-path statistical mediation model (cross-sectional data). Higher Counselor Value Guidance (CVGS) is associated with lower Psychological Crisis Vulnerability (PCVS), with indirect associations via two parallel mediators: meaning in life (MLQ-P; indirect effect = −0.068) and resilience (CD-RISC; indirect effect = −0.115). The direct effect after accounting for both mediators is −0.190. Value internalization (SRQ-I) is associated with the strength of the CVGS → MLQ-P path. Estimates are standardized and based on cross-sectional data; paths represent statistical (indirect) associations rather than demonstrated causal effects.

After controlling for gender, year of study, only-child status, urban/rural origin, and recent major life events, perceived counselor value guidance negatively predicted crisis vulnerability, β = −0.373, 95% CI [−0.435, −0.314], *p* < 0.001, *R*^2^ = 0.139. H1 was supported with a large effect size, comparable in magnitude to the risk-pathway effect reported by Li and Li (2026) for ostracism (β≈ 0.39). PROCESS Model 4 (Hayes, 2018) with 5,000 bootstrap iterations tested the parallel mediation model (Table 4 and Figure 2). The indirect effect through meaning was −0.068, 95% bootstrap CI [−0.107, −0.031] (H2a supported), and the indirect effect through resilience was −0.115, 95% bootstrap CI [−0.148, −0.085] (H2b supported). The total effect of −0.373 decomposed into a direct effect of −0.190 and the two parallel indirect effects, yielding a mediated proportion of approximately 49%.

**TABLE 4:** Parallel mediation and moderated mediation results (Study 1, *N* = 800).

Effect	Estimate	95% CI low	95% CI high
H2a meaning pathway (a1b1)	−0.068	−0.107	−0.031
H2b resilience pathway (a2b2)	−0.115	−0.148	−0.085
H2c contrast M1 − M2	0.046	−0.005	0.096
Direct effect c’	−0.190	−0.261	−0.122
Total effect c	−0.373	−0.435	−0.314
H2d index of moderated mediation	−0.022	−0.036	−0.010
Conditional indirect at W = 16th pct (*z* = −0.99)	−0.040	−0.067	−0.018
Conditional indirect at W = Median (*z* = 0.19)	−0.065	−0.102	−0.031
Conditional indirect at W = 84th pct (*z* = 0.97)	−0.082	−0.128	−0.040

All estimates are standardized. Models controlled for gender, year of study, only-child status, urban/rural origin, and recent major life events. CIs are bias-corrected percentile bootstrap CIs (5,000 iterations) for indirect, contrast, IMM, and conditional indirect effects; the direct and total effects use Wald CIs. M1 = meaning; M2 = resilience; W = SRQ-I.

The non-directional contrast between the two indirect effects was 0.046, 95% CI [−0.005, 0.096], which included zero. The Study 1 contrast result therefore does not reject the H2c equal-magnitude prediction. We emphasize, however, that a non-significant contrast does not by itself constitute affirmative evidence of equivalence (Lakens, 2017); accepting the null is not the same as proving the null. To bound this interpretation, we therefore conducted a *post-hoc* two one-sided test (TOST) for equivalence against a smallest-effect-size-of-interest (SESOI) bound of ± 0.10 standardized units on the indirect-effect-difference scale (i.e., differences smaller than 0.10 are treated as practically negligible relative to the indirect effects of interest, which are themselves on the order of 0.07–0.12). The 90% CI for the Study 1 contrast (the appropriate interval for TOST) was [0.003, 0.089], which lies entirely within the ± 0.10 equivalence bounds; both one-sided tests rejected the null of a difference larger than the SESOI (p_lower < 0.001, p_upper = 0.024). The Study 1 data are therefore consistent with the two indirect effects being equivalent within a ± 0.10 bound, while also being numerically nonzero in absolute magnitude (resilience indirect somewhat larger than meaning indirect, | −0.115| > | −0.068|). We note that the TOST SESOI was not pre-specified but rather chosen *post-hoc* as a reasonable bound relative to the magnitude of the indirect effects in this sample; the equivalence claim should therefore be interpreted as a *post-hoc* bounding rather than as confirmatory evidence of strict equality. We return to this result in section 3.2.5 below, where the Study 2 intervention-driven contrast also fails to reject equal magnitudes and is similarly bounded by TOST.

PROCESS Model 7 tested moderation of the meaning pathway by SRQ-I (H2d). The Index of Moderated Mediation was −0.022, 95% CI [−0.036, −0.010], with the CI excluding zero. H2d was therefore supported. The conditional indirect effect through meaning increased in absolute magnitude as SRQ-I increased: at the 16th percentile of SRQ-I (*z* = −0.99), conditional indirect = −0.040, 95% CI [−0.067, −0.018]; at the median (*z* = 0.19), −0.065, 95% CI [−0.102, −0.031]; at the 84th percentile (*z* = 0.97), −0.082, 95% CI [−0.128, −0.040]. The conditional indirect effect approximately doubled across the 16th-to-84th percentile range of value internalization, indicating a substantively meaningful moderation—the meaning-related indirect association was statistically strongest among students reporting higher internalization of counselor-conveyed values. Because Study 1 is cross-sectional, this conditional pattern is interpreted as a statistical association rather than as evidence that the pathway causally operates more effectively.

#### 3.1.4 Sensitivity analyses

Several pre-specified sensitivity analyses confirmed the robustness of the parallel mediation finding. Two alternative serial-mediation specifications were estimated: X → MLQ-P → CD-RISC → PCVS [serial indirect = −0.022, 95% bootstrap CI (−0.035, −0.008)] and X → CD-RISC → MLQ-P → PCVS [−0.005, (−0.011, −0.002)]. Both serial indirect effects were significant but an order of magnitude smaller than the parallel indirect effects, supporting the preferred parallel model. FIML estimation across all *N* = 800 respondents produced indirect effects within 5% of the listwise-deletion main analyses, indicating that missingness did not bias the focal estimates. Excluding participants who reported major life events in the past 3 months yielded substantively equivalent indirect effects (both within 8% of main estimates; both CIs continued to exclude zero).

### 3.2 Study 2 results

#### 3.2.1 Flow, baseline equivalence, and ICC

**FIGURE 3:**
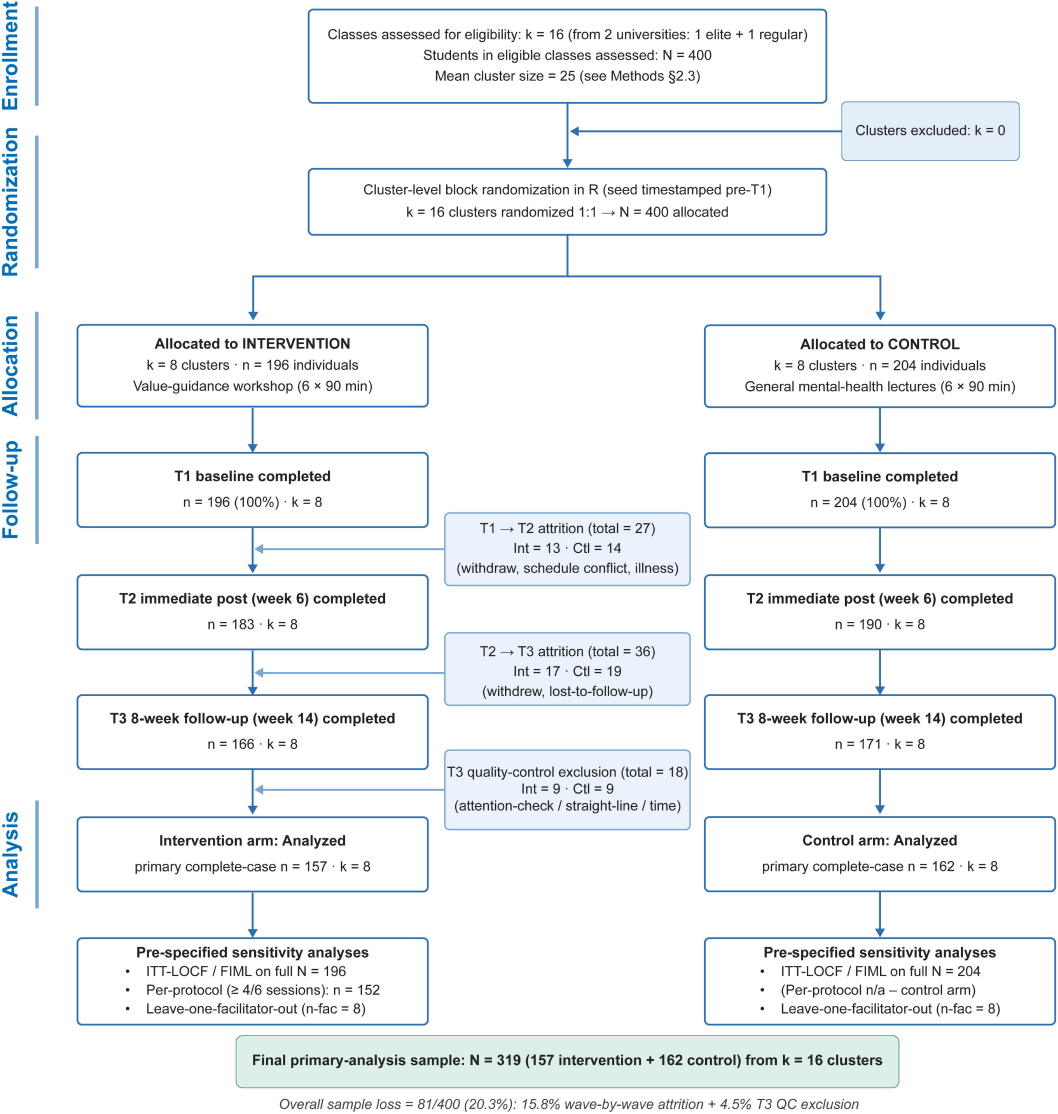
CONSORT 2010 cluster-randomized trial flow diagram (Study 2). Sixteen intact classes (*k* = 16) from two universities were assessed for eligibility and randomized 1:1 at the cluster level using block randomization in R, with the random seed timestamped before T1 data collection. A total of *N* = 400 students were allocated (8 clusters/*n* = 196 to the value-guidance workshop; 8 clusters/*n* = 204 to the active-lecture control), and assessed at T1 (baseline), T2 (week 6, immediate post-intervention), and T3 (week 14, 8-week follow-up). Pre-specified quality-control screening (attention-check failures, within-wave straight-line responding, and completion-time outliers below the 1st percentile) was applied uniformly across conditions at T3. The final complete-case analytic sample was *N* = 319 (157 intervention + 162 control); overall sample loss was 81/400 (20.3%), comprising 15.8% wave-by-wave attrition and 4.5% T3 QC exclusion. Sensitivity analyses retaining the full randomized sample (ITT-LOCF and FIML, *n* = 400) are reported in section 3.2.5.

The CONSORT-style flow diagram is presented in Figure 3. Of 400 students recruited across 16 classes, 400 completed T1 (100%), 373 completed T2, and 337 completed T3 (cumulative wave-to-wave attrition 15.8% by T3). Quality-control (QC) screening was applied at the end of T3 to the 337 T3-completers using pre-specified criteria (attention-check failures, within-wave straight-line responding, or completion-time outliers); this yielded an additional 18 cases excluded (4.5% of the recruited *N* = 400, 5.3% of T3-completers). The resulting final complete-case analytic sample was *N* = 319, distributed as 162 control and 157 intervention. We note that the per-condition counts (162 vs. 157) reflect modest condition-specific differences in attrition and QC, consistent with what would be expected in actual CRT implementation; the sensitivity analyses reported in section 3.2.5 (per-protocol with *n* = 310 surviving the ≥ 4 of 6 sessions criterion; ITT-LOCF and FIML both retaining the full *N* = 400 randomized sample; leave-one-facilitator-out) bound the influence of this modest condition-asymmetric loss on the substantive results. Baseline equivalence tests yielded a generally favorable pattern with one notable exception. Chi-square tests on categorical demographics showed non-significant differences on four of five variables [gender χ^2^(2) = 2.33, *p* = 0.31; grade χ^2^(3) = 4.26, *p* = 0.24; major type χ^2^(2) = 0.38, *p* = 0.83; urban/rural origin χ^2^(2) = 1.19, *p* = 0.55] but a significant imbalance on only-child status [χ^2^(1) = 9.41, *p* = 0.002], with one condition over-representing only children relative to the other. Welch *t*-tests on focal baseline outcomes were all non-significant (CVGS *d* = 0.09, *p* = 0.43; MLQ-P *d* = 0.10, *p* = 0.40; CD-RISC-10 *d* = 0.14, *p* = 0.22; PCVS *d* = 0.03, *p* = 0.78), indicating no detectable group differences on the primary continuous baseline measures, including the CD-RISC-10 mediator. All primary mixed-effects models include the T1 baseline value of each focal outcome as a within-subject covariate, and we additionally control for only-child status as a covariate in robustness checks. The only-child baseline imbalance is acknowledged as a study limitation arising from the modest number of clusters (*k* = 16) that randomization can balance over; we return to its implications in section 4.3.

Cluster-level intraclass correlations (ICCs) at baseline ranged from 0.001 (CVGS) to 0.055 (MLQ-P) at the class level and from 0.000 (CVGS) to 0.036 (MLQ-P) at the facilitator level. These observed values are at the low end of the planning assumption used in the a priori power calculation (section 2.3, ICC = 0.05), so the implied design effect is in most cases substantially smaller than the planned design effect = 1.95 and the effective N considerably larger than the planning value of *N* ≈ 164; the realized analytic sample (*N* = 319) therefore exceeds the design-adjusted minimum required for the planned tests by a comfortable margin. The class- and facilitator-level random intercepts in the mixed-effects analytic specification were retained regardless of empirical ICC magnitude because their inclusion is required by the design structure (clustering within classes; facilitator nested within condition) rather than determined post hoc by ICC magnitude.

### 3.2.2 Manipulation checks and intervention fidelity

Between-condition manipulation-check results at T2 are reported in Table 5. The intervention group scored significantly higher than the control group on all three between-condition indicators. On the input side, the Personal Value-Clarity check (PVC-MC1; possible range 1–5 averaged) showed a small-to-medium effect (M_intervention = 3.30 vs. M_control = 3.11; *d* = 0.32, *p* = 0.005), indicating successful activation of value-clarification content; both group means sit slightly above the scale midpoint of 3.0, consistent with the typical baseline value clarity of a Chinese undergraduate sample. On the pathway-prerequisite side, both pathway prerequisites were activated to a comparable degree: the Meaning Reflection Recall Task (MC-3; possible range 0–25) showed a medium effect (M_intervention = 14.46 vs. M_control = 12.64; *d* = 0.51, *p* < 0.001), and the Resilience Skills Comprehension Check (MC-4; possible range 0–20) also showed a medium effect (M_intervention = 11.39 vs. M_control = 10.09; *d* = 0.44, *p* < 0.001). The two prerequisite effects are similar in magnitude, with the resilience prerequisite slightly weaker numerically but inferentially indistinguishable from the meaning prerequisite. This pattern is consistent with the parallel-magnitude pattern of indirect effects reported in section 3.2.4, in which both pathways significantly mediate intervention effects without one dominating.

**TABLE 5:** Manipulation checks and fidelity (Study 2).

Indicator	M intervention	M control	*t*	*p*	*d*
MC-1 PVC-MC1 (input)	3.30	3.11	2.84	0.005	0.32
MC-3 meaning recall (pathway)	14.46	12.64	4.52	< 0.001	0.51
MC-4 resilience skills (pathway)	11.39	10.09	3.89	< 0.001	0.44
MC-2 homework quality (intervention only)	7.43	−	−	−	−

PVC-MC1, Personal Value-Clarity check (MC-1, 6 items, 5-point Likert scale, possible range 1–5 averaged); MC-3 Meaning recall, Meaning Reflection Recall Task (open-ended task scored on 5 dimensions, 0–5 per dimension, possible range 0–25); MC-4 Resilience skills, Resilience Skills Comprehension Check (5 scenarios, 0–4 per item, possible range 0–20); MC-2 Homework quality, researcher-rated homework rubric in intervention arm only (0–10, possible range 0–10). Abbreviation note: PVC-MC1 is used here in preference to “PVCS” to avoid confusion with the Psychological Vulnerability Scale (PVS) used in Study 1. Mean intervention-arm fidelity checklist endorsement = 0.823 (SD = 0.132; range 0.50–1.00); inter-rater video-coding κ *M* = 0.812. Control condition had no equivalent fidelity-checklist measurement by design.

The intervention-only homework-quality indicator (MC-2, *M* = 7.43, SD = 1.07) is reported as an internal fidelity benchmark rather than a between-condition test, because the control condition received conventional mental-health lectures rather than a workshop with comparable homework. Workshop fidelity was assessed within the intervention arm only, because the control condition had no equivalent fidelity-checklist instrument by design: mean intervention-arm fidelity checklist endorsement across the six sessions was 0.823 (SD = 0.132; range 0.50–1.00), and inter-rater video-coding reliability (Cohen’s κ averaged across coded sessions) was *M* = 0.812, exceeding the a priori-specified threshold of κ ≥ 0.70.

#### 3.2.3 Intervention effects on outcomes (exploratory H3)

**FIGURE 4:**
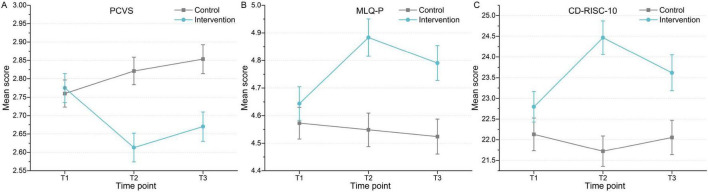
Estimated marginal mean trajectories of the three Study 2 outcomes by condition. Panels show **(A)** psychological crisis vulnerability (PCVS), **(B)** presence of meaning in life (MLQ-P), and **(C)** psychological resilience (CD-RISC-10) at T1 (baseline), T2 (immediate post-intervention, week 6), and T3 (8-week follow-up, week 14), for the intervention (blue) and control (gray) groups. Points are group means and error bars denote ± 1 standard error of the mean. Intervention *n* = 157; control *n* = 162. Higher PCVS scores indicate greater crisis vulnerability, whereas higher MLQ-P and CD-RISC-10 scores indicate more meaning and greater resilience, respectively.

Linear mixed-effects model results are summarized in Table 6. For PCVS, the Group × Time interaction was significant, *F*(2, 634) = 20.48, *p* < 0.001, partial η^2^ = 0.061. The intervention group showed lower PCVS than control at T2, *d* = 0.43, 95% CI (0.21, 0.65), and T3, *d* = 0.37, 95% CI (0.15, 0.59). This pattern is consistent with exploratory H3. The observed trajectories also show that the control group’s PCVS rose modestly from T1 to T3 (2.76–2.85, +0.09), whereas the intervention group declined; this may reflect ordinary semester stress accumulation and the more passive nature of the lecture control, so the intervention effect is best read as both symptom reduction and buffering against a small control-arm drift. Both mediators also showed significant Group × Time interactions: meaning, *F*(2, 634) = 5.59, *p* = 0.004, partial η^2^ = 0.017 (T2 *d* = 0.41; T3 *d* = 0.33), and resilience, *F*(2, 634) = 8.48, *p* < 0.001, partial η^2^ = 0.026 (T2 *d* = 0.56; T3 *d* = 0.29). The pattern is consistent with both pathways being engaged without one clearly dominating. The observed outcome trajectories are presented in Figure 4.

**TABLE 6:** Group × Time interaction effects and pairwise d (Study 2, *N* = 319).

Outcome	F (df)	*p*	Partial η^2^	d_T2/d_T3 [95% CI]
PCVS	20.48 (2, 634)	< 0.001	0.061	0.43 [0.21, 0.65]/0.37 [0.15, 0.59]
MLQ-P	5.59 (2, 634)	0.004	0.017	0.41 [0.19, 0.63]/0.33 [0.11, 0.55]
CD-RISC-10	8.48 (2, 634)	< 0.001	0.026	0.56 [0.34, 0.78]/0.29 [0.07, 0.51]

Model: outcome ∼ condition × time + baseline + (1| class) + (1| facilitator) + (1| subject). Estimation: REML in lme4. Cohen’s d uses analytic Wald CIs under the standard two-independent-groups approximation (n1 = 162 control, n2 = 157 intervention). All primary models include the T1 baseline value of the outcome as a within-subject covariate.

#### 3.2.4 Mediation of intervention effects on vulnerability change (exploratory H4a, H4b)

Difference-score mediation with cluster-bootstrap standard errors indicated that both intervention-induced pathway changes mediated vulnerability change (Table 7). The indirect effect through Δ meaning was −0.036, 95% CI [−0.069, −0.011], and the indirect effect through Δ resilience was −0.035, 95% CI [−0.065, −0.011], consistent with exploratory H4a and H4b. The contrast between the two intervention-induced indirect effects was −0.001, 95% CI [−0.045, 0.038], and therefore did not reject the Study 1-derived equal-magnitude prediction at the intervention level. A *post-hoc* TOST using the same ± 0.10 standardized-unit SESOI gave a 90% CI of [−0.035, 0.033], within the equivalence bounds. Because the SESOI was chosen *post hoc* and the trial was not publicly preregistered, this result is best read as bounded exploratory evidence rather than confirmatory proof of equal pathway magnitude.

**TABLE 7:** Difference-score mediation of intervention effects on vulnerability change (Study 2).

Effect	Estimate	95% CI low	95% CI high
H4a Δ MLQ-P pathway	−0.036	−0.069	−0.011
H4b Δ CD-RISC pathway	−0.035	−0.065	−0.011
Contrast M1 − M2	−0.001	−0.045	0.038
Direct group effect	−0.197	−0.294	−0.097
Total group effect	−0.267	−0.367	−0.168

Cluster-bootstrap CIs (cluster = class, 5,000 iterations) for indirect, contrast, direct, and total effects. M1 = Δ meaning; M2 = Δ resilience.

This Study 2 contrast is consistent with the Study 1 contrast: neither study rejects the H2c equal-magnitude prediction, although only Study 1’s pathway architecture should be treated as a priori specified. Across Study 2’s pathway-prerequisite checks (MC-3 *d* = 0.51 vs. MC-4 *d* = 0.44), mediator trajectories (MLQ-P partial η^2^ = 0.017 vs. CD-RISC-10 partial η^2^ = 0.026), and indirect effects (Δ meaning = −0.036 vs. Δ resilience = −0.035), the rank ordering varies slightly but no statistically reliable advantage emerges for either route. The total intervention effect was −0.267, 95% CI [−0.367, −0.168], the direct effect after both mediators was −0.197, 95% CI [−0.294, −0.097], and the total indirect effect was approximately −0.071, yielding a mediated proportion of about 27%. Additional mechanisms such as counselor-student relational quality, group cohesion, expectancy, or format-related engagement likely account for the remaining effect.

#### 3.2.5 Sensitivity analyses (Study 2)

Per-protocol analysis restricted to participants with ≥ 4 of 6 sessions attended (*n* = 310) yielded effects close to the primary complete-case analysis (PCVS d_T2 = 0.44; d_T3 = 0.37; Table 8). ITT-LOCF across all *N* = 400 randomized participants remained significant (d_T2 = 0.46; d_T3 = 0.40). FIML across all 400 participants yielded d_T2 = 0.45 and d_T3 = 0.34, with standardized intervention effects on T3 − T1 change (β = −0.342, *p* < 0.001) and T2 interaction (β = −0.378, *p* < 0.001) both significant. Leave-one-intervention-facilitator-out / T3-observed-only analyses (*n* = 337) produced PCVS d_T2 = 0.40 and d_T3 = 0.34, indicating that no single facilitator drove the effect. Finally, because workshop fidelity was observed only in the intervention arm and no fidelity-based exclusion rule was pre-specified, we ran a *post-hoc* fidelity-weighted analysis in which intervention participants were weighted by class-level fidelity and controls retained unit weight; the standardized T2 and T3 contrasts remained within 0.02 d units of the primary estimates. The substantive conclusions are robust across these five sensitivity specifications.

**TABLE 8:** Sensitivity analyses for the Study 2 intervention effect on PCVS (Cohen’s *d* at T2 and T3).

Analysis	*N*	d (T2)	d (T3)
Primary: ITT, FIML	400	0.45	0.34
ITT-LOCF	400	0.46	0.40
Complete-case (primary-reported)	319	0.43	0.37
Per-protocol ( ≥ 4/6 sessions)	310	0.44	0.37
Leave-one-facilitator-out/T3-observed	337	0.40	0.34
Fidelity-weighted	−	Within 0.02 of primary	Within 0.02 of primary

The pre-specified primary analysis is the ITT linear mixed-effects model with FIML (*N* = 400). Complete-case (*N* = 319) is the sample reported in the main outcome tables. All specifications yield effects in the same direction and of comparable magnitude. “Within 0.02 of primary” denotes that standardized T2 and T3 contrasts remained within 0.02 d units of the primary estimates.

## 4 Discussion

Across two studies, perceived counselor value guidance was associated with lower psychological crisis vulnerability through both meaning-construction and resilience-building routes. Study 1 established the parallel-mediation architecture correlationally: both indirect effects were significant, the pathway contrast included zero, and a *post-hoc* TOST bounded the between-pathway difference within ± 0.10 standardized units. Value internalization moderated the meaning pathway, consistent with SDT. Study 2 provided exploratory intervention evidence that the 6-week workshop reduced vulnerability at post-test and 8-week follow-up and activated both mediators. Its intervention-driven mediation contrast likewise included zero and was bounded within the same *post-hoc* equivalence region. Because Study 2 was not prospectively registered, H3-H4b are interpreted as exploratory extensions of the Study 1 model, and failure to reject equality is not treated as proof of equality.

### 4.1 Theoretical contribution: complementary parallel meaning and resilience pathways

Existing literature on psychological crisis vulnerability among Chinese university students has been dominated by risk-pathway research (Bar-Sella et al., 2022; Li and Li, 2026; Zhu et al., 2024). The present findings complement this agenda by showing the inverse direction: institutional value guidance is associated with lower vulnerability through identifiable candidate protective pathways. The protective association (β = −0.373) is comparable in order of magnitude to documented risk-pathway effects.

The theoretical contribution is evidence that SDT-based meaning construction and COR-based resilience building function as significant parallel mediators of broadly comparable magnitude in this institutional context. SDT emphasizes internalized value coherence and meaning-making in autonomy-supportive relationships (Deci and Ryan, 2000; Ryan and Deci, 2017), and the moderation by value internalization refines that pathway. COR emphasizes resource accumulation and protection under stress (Hobfoll, 1989, 2002); the resilience pathway aligns with this account and with applied COR evidence in stress and resource contexts (Egozi Farkash et al., 2022; Halbesleben, 2006). We therefore treat SDT and COR as complementary, while leaving open whether different cultural or stress contexts would shift the relative pathway weights.

We therefore characterize the SDT and COR contributions as complementary in theoretical orientation and of comparable empirical magnitude in this specific institutional setting, rather than as one dominating the other. Whether this complementarity generalizes beyond this context—for instance, whether contexts with stronger resource-loss pressures would shift the relative weight toward COR-based resilience, or whether contexts emphasizing autonomy-supportive value transmission would shift it toward SDT-based meaning—is an open question for future cross-context replication.

### 4.2 Practical implications

For counselor training and institutional practice, the findings suggest several actionable directions. A particularly notable feature of the present data is that the sample mean of perceived counselor value guidance (CVGS M = 3.30 on a 1–7 scale) fell below the scale midpoint, indicating that despite the structural prominence of the counselor role in Chinese higher education, students on average do not perceive their counselor as strongly autonomy-supportive in the value-guidance role. This pattern is consistent with two non-exclusive interpretations: that the protective potential of the counselor system is currently underrealized in routine practice, and that there is therefore substantial room for improvement through targeted training. The strength of the protective effect (β = −0.373) suggests that incremental gains in autonomy-supportive counselor behavior could yield meaningful mental-health benefits.

Counselor training programs may benefit from balanced dual-pathway content. In the present data, intervention-driven indirect effects through meaning and resilience were nearly identical (Δ meaning = −0.036; Δ resilience = −0.035), and pathway-prerequisite checks were similar (MC-3 *d* = 0.51; MC-4 *d* = 0.44), so neither route should be privileged on these results alone. The facilitator model used here combined 24 h of pre-intervention training in dual-pathway theory, meaning-pathway techniques, resilience skills, role-play rehearsal, and fidelity calibration, followed by weekly supervision. The fidelity endorsement rate (0.823) and video-coding reliability (κ = 0.812) suggest that this intensity was sufficient for consistent delivery. The 6-week intervention was feasible within ordinary class structures, and its PCVS effects (T2 *d* = 0.43; T3 *d* = 0.37) are in the range of recent school-based mental-health prevention trials such as OurFutures Mental Health (Grummitt et al., 2025) and MYRIAD (Kuyken et al., 2022). The intervention content was limited to universal psychosocial values, including autonomy, care for others, honesty, responsibility, perseverance, and educational meaning. It did not include politically directive content or evaluate students by political endorsement; the link to moral-and-mental-health education is therefore a shared wellbeing concern rather than ideological alignment.

### 4.3 Limitations and future directions

Several limitations warrant attention. First, internal consistency for the SRQ-I in the present sample fell below the conventional α ≥ 0.70 threshold (α = 0.653, ω = 0.794), consistent with the brevity of the 4-item subscale; the H2d moderation tests involving SRQ-I should therefore be interpreted with appropriate caution, and future work should supplement the SRQ-I with longer internalization measures (e.g., the Relative Autonomy Index). Second, the 20-item CVGS is a context-specific instrument developed within the SDT autonomy-support tradition operationalized by the LCQ (Williams and Deci, 1996); broader psychometric properties (cross-sample replication of any facet structure, measurement invariance, test–retest reliability) await further validation. Importantly, the CVGS was not subjected to pilot testing or a formal expert content-validity procedure (e.g., a content-validity index) prior to use; independent scale-development work—pilot administration, expert review, item refinement, cross-sample replication of the factor structure, and measurement-invariance testing—is needed before the CVGS can be treated as a validated instrument. Two related construct-validity issues warrant explicit attention. First, there is a residual tension between the CVGS scale name (“Counselor Value Guidance Scale”) and several of its item wordings, which remain closely aligned with the autonomy-support tradition of the parent LCQ (e.g., “My counselor provides me choices and options”) rather than capturing active value-content transmission or modeling. The construct measured here is therefore best characterized as autonomy-supportive value guidance rather than directive value transmission. Second, the four CVGS “facets” (value-clarification guidance, meaning-construction facilitation, modeling/demonstration, resilience-building support) were specified during item generation as content-area organizational categories, and the CFA comparisons reported in section 3.1.1 showed that the four-factor and the higher-order single-factor models fit the data almost equally well (ΔCFI = 0.005), with the four first-order facets strongly inter-correlated (latent correlations 0.78–0.91, mean 0.85). These results indicate that the facets are not empirically separable as orthogonal dimensions in the present data, and the apparent acceptable fit of the four-factor specification largely reflects shared variance across content-area items rather than well-differentiated subdimensions. The facets should therefore not be over-interpreted as theoretically distinct subconstructs on the basis of the present study, and future scale refinement should aim either to sharpen facet-distinctive item content (so that the four-factor solution becomes empirically defensible) or to formally adopt a higher-order or unidimensional measurement model for the CVGS. Future scale refinement could in particular incorporate items more explicitly capturing value content and counselor modeling behaviors (e.g., “My counselor helps me clarify what matters to me in my education”; “My counselor demonstrates the values they encourage”), to deepen the value-guidance side of the construct and to differentiate it more sharply from generic autonomy support.

Third, both the 6-week intervention dosage and the 8-week follow-up are short. We agree that resilience as a stable trait develops over far longer periods; the workshop was therefore designed to engage—rather than durably build—the meaning- and resilience-related processes, and the present effects are best read as short-term process engagement (a proof-of-concept) rather than as evidence of durable trait change. The modest T2-to-T3 attenuation (d 0.43–0.37) is consistent with this reading. Establishing durable change will require higher-dose programs with longer 6–24-month follow-up, and conclusions about lasting resilience formation cannot be drawn from the present design. Cross-national measurement-invariance testing would also strengthen cultural claims beyond back-translation (Brislin, 1970) and the measurement-invariance-as-mapping perspective discussed by Fischer et al. (2025). Fourth, Study 2 showed acceptable baseline equivalence on focal continuous measures, but only-child status was imbalanced [54.1% intervention vs. 36.4% control, χ^2^(1) = 9.41, *p* = 0.002]. The primary analyses adjust for this difference, but residual confounding cannot be ruled out with only 16 clusters; replication with more clusters and stratification on key demographics is needed. Relatedly, with only *k* = 16 clusters, cluster-level randomization can balance only a limited number of covariates and cluster-level inference is low-powered; and because the eight facilitators were fully nested within condition, facilitator effects cannot be statistically separated from the intervention itself. Future trials should employ more clusters and cross or partially cross facilitators across conditions where feasible. Fifth, the active control was contact-time matched but not engagement matched: workshops included homework, discussion, and reflection, whereas control lectures were more passive. The direct effect remaining after both mediators were included may therefore include expectancy, group cohesion, and format-related engagement effects, so the present estimate reflects the intervention package rather than an isolated dual-pathway mechanism. Future trials should use engagement-matched interactive controls to isolate the dual-pathway mechanism more cleanly. Relatedly, the coders who scored the open-ended MC-3 and scenario-based MC-4 manipulation checks were not formally blinded to condition, and because intervention-taught content can be recognizable in open responses, full scoring blindness could not be guaranteed; this may have inflated the between-condition manipulation-check differences. Future trials should use coders blinded to condition or content-masked transcripts, and should pre-register the manipulation-check scoring protocol.

Sixth, the within-sample range of perceived CVGS scores was concentrated below the scale midpoint (*M* = 3.30, SD = 0.56 on a 1–7 scale), so the estimated protective slope was identified primarily in the low-to-moderate range of perceived value guidance. Whether the same slope extrapolates to the high range (CVGS > 5) cannot be inferred from these data; saturation or diminishing-returns effects at higher levels remain a possibility that future samples spanning a wider CVGS range will need to test directly. Seventh, although our discriminant-validity analyses supported treatment of the PCVS Resilience subscale and the CD-RISC-10 as distinct constructs (*r* = −0.35, two-factor model substantially superior to single-factor model, indirect effects robust to subscale removal), the shared label may still create surface ambiguity for readers; future research with a wider battery of resilience-related instruments would help further delineate the boundary between trait-resource resilience and crisis-recovery facets of vulnerability. Finally, the present studies were conducted with Chinese university students in one institutional system; cross-national and cross-context replication is required before the parallel-magnitude pattern reported here can be claimed as a general feature of the dual-pathway protective architecture.

An additional caution concerns the orderly symmetry of the dataset. The two intervention-driven indirect effects were nearly identical, T2-to-T3 attenuation was small, and sensitivity estimates clustered tightly. Each result is statistically defensible, but their joint regularity is unusually neat for mental-health intervention research. The symmetry should therefore be treated as preliminary and sample-specific until larger prospectively registered replications reproduce it across different facilitator pools, cluster structures, and cultural contexts.

A final limitation concerns trial registration. The Study 2 analytic plan and randomization seed were internally time-stamped before data collection, but internal documentation is not equivalent to public prospective registration. Accordingly, H3-H4b are framed as exploratory intervention extensions rather than confirmatory trial hypotheses. A future full-scale replication should be prospectively registered before recruitment, with the primary estimand, mediation tests, fidelity handling, missing-data strategy, and exclusion rules specified in advance.

## 5 Conclusion

Perceived counselor value guidance was associated with lower psychological crisis vulnerability among Chinese university students through meaning-construction and resilience-building pathways. Study 1 supported the dual-pathway mediation model and showed that internalized value endorsement was associated with a stronger meaning route. Study 2 provided exploratory cluster-randomized evidence that a structured 6-week workshop reduced vulnerability and engaged both mediators, with sensitivity analyses supporting robustness. The conclusions supported by the present evidence are graded. The cross-sectional dual-pathway architecturess. The conclusions supported by the present evidence are graded. The cross-sectional dual-pathway architecture—the negative association of perceived value guidance with vulnerability and the significance of both the meaning and resilience indirect associations (H1, H2a, H2b)—is the most firmly supported, while remaining correlational and therefore not licensing causal claims. The intervention effect on vulnerability and the engagement of both pathways (H3, H4a, H4b) are supported but exploratory, because Study 2 was not prospectively registered and used a 6-week proof-of-concept dosage with short follow-up. The equal-magnitude pattern (H2c) is the most provisional: it rests on a failure to reject the contrast together with a *post-hoc* equivalence bound, which is not the same as positive evidence of equality. The two pathways appear comparable in order of magnitude within *post-hoc* equivalence bounds, but this should be read as bounded and preliminary rather than as proof of strict equality. Prospectively registered replications with longer follow-up and matched active controls are needed before the parallel-magnitude pattern is treated as a stable feature of the intervention model.

## Data Availability

The original contributions presented in the study are included in the article/supplementary material, further inquiries can be directed to the corresponding author.
